# A COVID-19 outbreak with a high attack rate among inpatients in a psychiatric hospital in Wenzhou, China

**DOI:** 10.3205/dgkh000498

**Published:** 2024-10-02

**Authors:** Yiwei Zhou, Xingbao Lin, Yu Huang, Chunhua Wu, Jun Li, Jieru Huang, Zumu Zhou

**Affiliations:** 1 Business School, University of Shanghai for Science and Technology, Shanghai, China; 2 School of Intelligent Emergency Management, University of Shanghai for Science and Technology, Shanghai, China; 3 Smart Urban Mobility Institute, University of Shanghai for Science and Technology, Shanghai, China; 4 The Affiliated Kangning Hospital of Wenzhou Medical University, Zhejiang Provincial Clinical Research Center for Mental Disorders, Wenzhou, China; 5 Wenzhou Center for Disease Control and Prevention, Wenzhou, China

**Keywords:** COVID-19, outbreak, cluster, psychiatric hospital

## Abstract

**Background:**

Since the emergence of COVID-19, China has taken strict prevention and control measures against the disease. At the end of 2022, when the government optimized and relaxed the COVID-19 prevention and control policy, a new wave of the epidemic appeared. This paper reports an outbreak of COVID-19 among inpatients in a psychiatric hospital in Wenzhou, China.

**Methods:**

The case definition of COVID-19 was established. Throat swab specimens were collected and examined by RT-PCT. Questionnaires were distributed to hospitalized patients. Survey data were collected and analyzed.

**Results:**

Of 902 inpatients in our hospital, 762 became infected with COVID-19, an attack rate of 84.5%. The outbreak occurred from 10 December, 2022 to January 18, 2023. Twenty patients died, yielding a case fatality rate of 2.6%. Most of the dead were among elderly people with underlying diseases, and 19 of the deceased were over 65 years old.

**Conclusion:**

Our investigation showed that the outbreak of COVID-19 among hospitalized patients had the characteristics of high incidence, strong infectivity, and rapid transmission. Infectious sources were introduced into the hospital from the community and spread within the hospital, resulting in an outbreak. In addition, special attention should be paid to elderly people with underlying diseases when treating patients with COVID-19 in the future.

## Zusammenfassung

**Hintergrund:**

Seit dem Auftreten von COVID-19 hat China strenge Präventions- und Kontrollmaßnahmen gegen die Krankheit ergriffen. Ende 2022, als die Regierung die Präventions- und Kontrollmaßnahmen für COVID-19 optimierte und lockerte, trat eine neue Welle von Epidemien auf. Dieser Artikel berichtet über einen Ausbruch von COVID-19 bei stationären Patienten in einem psychiatrischen Krankenhaus in Wenzhou, China.

**Methode:**

Die Falldefinition von COVID-19 wurde festgelegt. Es wurden Rachenabstriche entnommen und mittels RT-PCT untersucht. An die Krankenhauspatienten wurden Fragebögen verteilt. Die Erhebungsdaten wurden gesammelt und analysiert.

**Ergebnisse:**

Es wurden 920 Patienten stationär im Krankenhaus aufgenommen. Bei 762 Patienten trat COVID-19 auf, d.h. die Ansteckungsrate betrug 84,5%. Der Ausbruch erfolgte vom 10. Dezember 2022 bis zum 18. Januar 2023. Es ereigneten sich 20 Todesfälle mit einer Sterblichkeitsrate von 2,6%. Bei den meisten Verstorbenen handelte es sich um ältere Menschen mit Grunderkrankungen; 19 Verstorbene waren über 65 Jahre alt.

**Schlussfolgerung:**

Unsere Untersuchung ergab, dass der Ausbruch von COVID-19 bei Krankenhauspatienten durch eine hohe Inzidenz, eine starke Infektiosität und eine schnelle Übertragung gekennzeichnet war. Die Erreger wurden aus der Bevölkerung in das Krankenhaus eingeschleppt und verbreiteten sich innerhalb des Krankenhauses, was zu einem Ausbruch der Krankheit führte. Im Rahmen des Ausbruchmanagements sollte bei der Behandlung von Patienten mit COVID-19 in Zukunft besonders auf ältere Menschen mit Grunderkrankungen geachtet werden.

## Introduction

Since the emergence of COVID-19, outbreaks and epidemics of varying severity have occurred in various countries [1], [2]. The COVID-19 outbreaks have sometimes been reported in nursing homes, medical institutions, schools, shopping malls, families, fitness centers, and public venues [2], [3], [4], [5], [6], [7], [8]. Since the outbreak of COVID-19 in Wuhan, China has taken strict prevention and control measures, and the epidemic has been effectively controlled in scale and intensity. However, due to the close relations between China and other countries, the SARS-COV-2 mutant strains have been continuously imported, and sporadic cases or clusters of COVID-19 have often occurred throughout the country; for instance, Wenzhou has also experienced outbreaks of COVID-19 in family clusters and shopping malls [2], [5], as well as in many other regions and other medical institutions. Recently, a COVID-19 outbreak occurred in a psychiatric hospital in Wenzhou, China, at a time when the national measures for COVID-19 prevention and control were being adjusted, optimized, and relaxed. Immediately after the outbreak, we investigated the clinical and epidemiological characteristics of this COVID-19 outbreak to provide a basis for future responses to COVID-19 and similar outbreaks. 

## Methods

### Case definition

COVID-19 cases hospitalized between December 8, 2022 and January 18, 2023 with a history of exposure to COVID-19 patients and with acute onset of fever and cough (ILI) or acute onset of three or more of the following symptoms: fever, cough, malaise, muscle aches, nasal congestion, runny nose, sore throat, decreased sense of smell and taste, diarrhea, and conjunctivitis [9]; or pulmonary imaging exhibited signs of pneumonia; or positive PCR test.

### Questionnaire

Using a uniformly designed questionnaire, a nurse manager on each ward was responsible for the survey on their respective ward, asking questions, and recording the incidence and associated conditions of each inpatient between December 1, 2022, and January 30, 2023, including age, sex, time of onset, clinical symptoms, vaccination status, laboratory tests, chest CT and/or radiographic findings. Questionnaires were completed and returned. The collected data were analyzed using Excel software. All case investigations were performed by epidemiologists.

Laboratory tests: Throat swabs were collected from cases early in the outbreak and SARS-CoV-2 nucleic acid was detected by RT-PCR. Some outbreak cases were also selected for detection of SARS-CoV-2 antigen.

### Statistical method

Statistical analysis was performed using Excel software. The Chi-square test was used for categorical variables. Statistical significance was set at P<0.05. 

### Ethical approval

The present survey was approved by the Medical Ethics Committee of The Affiliated Kangning Hospital of Wenzhou Medical University, Wenzhou, China (No 2023017). This study was conducted in accordance with the Declaration of Helsinki and Good Clinical Practice. All participants gave informed consent. Before the survey was conducted, each patient was informed that the information collected in this survey would be kept confidential and would not be disclosed; the data were kept by a specifially designated person. The purpose and scope of the study were explained in the questionnaire at the beginning of the survey. A few sentences about voluntary informed consent were added at the beginning of the questionnaire. The patient was aware of the use of this information; if the patient was not willing to comply with the survey, they could not complete the questionnaire.

## Results

### Overview

The psychiatric hospital is a tertiary class-A hospital with 1,200 beds and more than 1,100 employees,integrating medical treatment, teaching and rehabilitation. It has an inpatient and an outpatient department. The inpatient department has 19 wards, including comprehensive ward, child and adolescent psychology ward, sleep medicine ward, clinical psychology ward, early mental intervention ward, psychosomatic disorders ward, emotional disorders ward, behavioral medicine ward, memory disorders ward, geriatric psychiatry ward, women’s psychiatry ward, general psychiatry ward, and special care ward. Patients in the special care ward are usually widowed elderly people with long hospital stays and financial difficulties, who have received little care from their families and belong to vulnerable groups.

### Distribution over time

**Figure 1:**
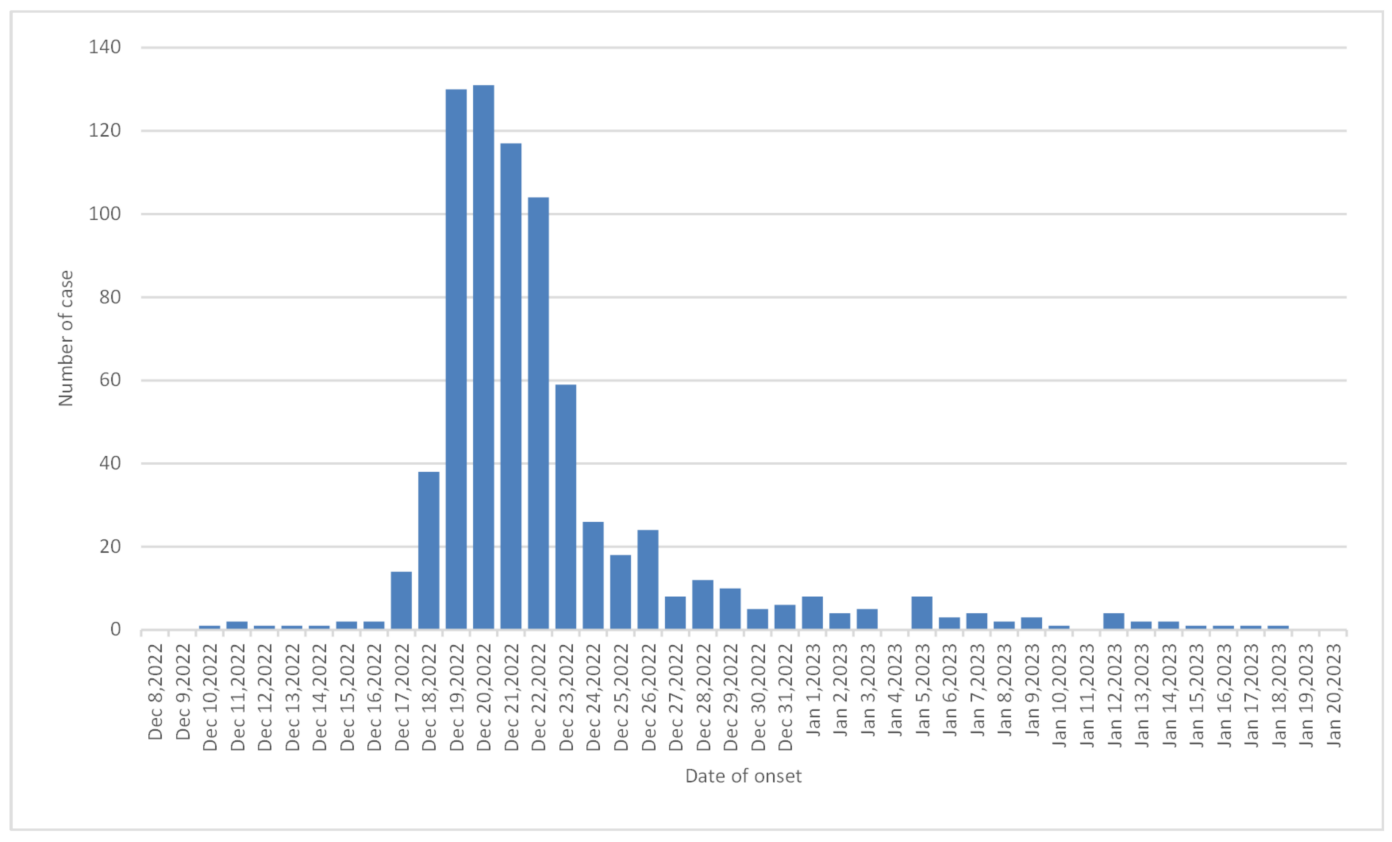
Temporal distribution of 762 COVID-19 cases in the psychiatric hospital from December 2022 to January 2023

During the COVID-19 outbreak, 902 patients were hospitalized between December 10, 2022, and January 18, 2023, of whom 762 became ill (attack rate 84.48%). The first case occurred on December 10, 2022, followed by a succession of sporadic cases until December 16, with a gradual increase after December 17 and more than 100 cases per day from December 19 to 22, reaching peak incidence. This was followed by a gradual decrease until January 18, 2023, when the outbreak was terminated. The number of cases from December 19 to 22 accounts for 63.25% of the total number of cases in this hospital. For temporal distribution of COVID-19 cases in the psychiatric hospital, see Figure 1 (Fig. 1).

### Distribution of cases by ward

The infection rate of COVID-19 was 100% in the comprehensive ward, child and adolescent psychiatry ward, clinical psychology ward, memory disorders ward I, and memory disorders ward III, while the lowest incidence rate was 58.90% in the women’s psychiatry ward. The difference in COVID-19 attack rates between wards was significant (χ^2^=115.02, P<0.01; Table 1 (Tab. 1)). 

### Attack rate by population

Among 902 subjects, 762 cases were affected. The overall attack rate of COVID-19 was 84.5% with males making up 88.39% of these, and females 78.1%, a highly stastistically significant difference (χ^2^=17.25, P<0.01). In terms of age, the highest attack rate was 96.3% in the group aged 75–79 years and the lowest was 76.5% in the group aged 30–39 years. When these cases were divided into groups ≤59 years of age and ≥60 years of age, the attack rates were 80.3% and 88.2% respectively. (χ^2^=4.18, P<0.05, Table 2 (Tab. 2)).

### Clinical manifestations

Fever was proportionally higher than other symptoms, accounting for 96.1%; the other symptoms in decreasing order were dry cough, malaise, nasal congestion, sore throat and runny nose, accounting for 75.6%, 62.1%, 50.1%, 38.6% and 38.3%, respectively, while the proportion of loss of taste or smell, myalgia, diarrhea and conjunctivitis was lower (Table 3 (Tab. 3)).

### Cases of death

Between December 21, 2022 and January 20, 2023 out of the 762 COVID-19 cases, there were 20 deaths, 13 male and 7 female (a case fatality rate of 2.6%). With the exception of one 55-year-old patient, the remaining 19 deaths were over 65 years of age (of which three were 65–69, seven were 70–79, six were 80–89 and three were ≥90 years of age). Seventeen (17) deaths were due to pulmonary infection, one to myocarditis, one to uremia, and one to gastrointestinal bleeding. All three patients with myocarditis, uremia, or gastrointestinal bleeding were nucleic-acid positive by RT-PCR. Complications and/or comorbidities of these deaths included mental disorders (e.g, Alzheimer’s disease, schizophrenia, epileptic psychosis, epilepsy, delirium, vascular dementia, psychotic disorder, etc.) and chronic obstructive pulmonary disease (COPD), hypertension, type 2 diabetes mellitus, anemia, renal insufficiency, sequelae of cerebral infarction, osteoporosis, lacunar cerebral infarction, chronic hepatitis B, cholecystitis, chronic bronchitis, and other diseases.

### Vaccination status of inpatients

After the COVID-19 outbreak, the COVID-19 vaccination status of hospitalized inpatients was investigated. Among the 902 cases investigated, 141 cases (15.6%) had been vaccinated with COVID-19 vaccine, including 11 cases (1.2%) with 1 dose of vaccine, 48 cases (5.3%) with 2 doses, 82 cases (9.1%) with 3 doses or more, 752 cases (83.4%) without vaccination, and 9 cases (1%) with unknown vaccination status. Among the 286 patients in the special care ward, only 1 case was fully vaccinated, i.e., the full vaccination rate was 0.4%; among the 616 cases in the non-special care ward, 139 cases were fully vaccinated, i.e., the full vaccination rate was 22.6%. The difference in the full vaccination rate between the special care wards and non-special care wards was highly significant (χ^2^=67.14, P<0.01). There were 130 cases with full vaccination (2 or more doses), of which 85 cases were morbid, an attack rate of 65.4%; 752 cases were not vaccinated, of which 662 cases were confirmed with COVID-19, i.e., an attack rate of 88%. The difference in attack rate between the full vaccination group and the non-vaccination group was highly significant (χ^2^=43.85, P<0.01, Table 4 (Tab. 4)).

### Prevention and control measures

After the outbreak, the hospital reacted quickly and took various prevention and control measures: doctors and nurses were instructed to closely monitor the change of the patient's condition and make all preparations for treatment; at the same time, personal protection to prevent and control the infection of medical personnel was comprehensively implemented; the hospital's emergency plan was activated. The laboratory and radiology departments worked overtime to produce test results as quickly as possible to provide physicians with timely diagnosis and treatment advice; patients were isolated; infected areas were disinfected; windows were opened for ventilation; and hand hygiene was reinforced. Staff access management was optimized and internal zoning management was implemented based on facility conditions.

## Discussion

Over the past three years, governments at all levels have attached great importance to strict prevention and control measures against the COVID-19 pandemic and have expended a great deal of human and material resources to prevent and control the occurrence and spread of COVID-19, which has affected social development and economic operation as well as people’s daily lives, while effectively controlling the pandemic. It would be impractical and difficult to maintain these stringent prevention and control measures in the long term. In addition, in view of the mass vaccination with COVID-19 vaccine, the mutation of the SARS-COV-2 strain, the results of monitoring the predominant strain of SARS-COV-2, the existing experience in the prevention and control of COVID-19 at home and abroad, and the available medical and health resources, China has recently adjusted and optimized the prevention and control measures for COVID-19.

While the COVID-19 prevention and control measures were being adjusted and optimized, COVID-19 outbreaks occurred in most areas of China, and COVID-19 outbreaks also occurred in many local medical institutions. As a specialized hospital for the treatment of psychiatric and elderly patients, despite having taken many measures to prevent and control COVID-19, it was not spared from a COVID-19 outbreak. This survey confirmed the outbreak of COVID-19 among hospitalized patients in a psychiatric hospital based on epidemiology, clinical characteristics, and laboratory tests. The investigation showed that the source of infection of the outbreak was from the community. There were a large number of infectious sources in the community, enabling the virus to spread to the hospital. In this period of policy adjustment, the number of COVID-19 cases occurring in the community has increased rapidly, and it was difficult to control them effectively in a short period of time. As some outpatients carring SARS-CoV2 from the community came to our hospital for consultation and treatment, the virus was brought into the hospital and spread widely there, forming an outbreak. The present investigation showed that the first COVID-19 case in the COVID-19 outbreak in the psychiatric hospital occurred on December 10, 2022. According to a follow-up investigation, RT-PCR-positive nucleic-acid cases were identified in the outpatient laboratory of the hospital on December 8 and 9. In fact, before the outbreak, there were also some sporadic cases of COVID-19 in the local community where some outbreak-associated patients lived. Our survey showed that COVID-9 cases in the hospital were prevalent in the latter phase of the spread of the disease in the community, which was consistent with the results reported by Imamura et al [10]. However, the underlying mechanisms for the trend might differ between different medical institutions and countries.

In addition, the cause of outbreaks among psychiatric hospital inpatients may be related to the following factors. Psychiatric and elderly patients are highly susceptible to COVID-19 [11], [12], [13], and this high susceptibility is closely related to high infectivity and severe consequences. In addition, the patients admitted to this hospital are often older. The proportion of elderly and mentally ill patients in the hospital is high, and there are many patients with underlying diseases. Their immunity is weaker than that of younger, healthier individuals. In addition, the rate of COVID-19 vaccination among the elderly patients is very low; all of these factors contributed to the outbreak.

Ng et al. [14] reported that the attack rate of patients in six COVID-19 outbreaks in hospitals was 21.4±16.3%. In the United Kingdom, 5%–7% of COVID-19 cases have been reported to be associated with nosocomial infections [12]. A prospective study in a teaching hospital in London showed that 15% of COVID-19 cases were definitely or probably associated with nosocomial infections [15]. The present investigation showed an attack rate of 84.47% among hospitalized patients, which is higher than that reported by other investigators [12], [14], [15]. Ng et al. [14] reported 28 COVID-19 outbreaks at various hospitals, with a case fatality rate of 15.0±20.7%. Abbas et al. [15] reported that a case fatality rate of 36% for COVID-19 patients at the teaching hospital in London. In another study of COVID-19 outbreak, the COVID-19 case fatality rate was as high as 50.0% in patients with underlying blood disorders [14]. The present study showed that the COVID-19 case fatality rate among psychiatric hospital inpatients was 2.6%, which was lower than that reported in other studies [14], [15]. Additionally, The current investigation of the COVID-19 outbreak showed that most of these deaths were of patients with underlying medical conditions, including mental disorders (e.g., Alzheimer’s disease, schizophrenia, epileptic psychosis, epilepsy, delirium, vascular dementia, psychotic disorders, etc.), chronic obstructive pulmonary disease (COPD), hypertension, type 2 diabetes mellitus, anemia, renal insufficiency, cerebral infarction sequelae, osteoporosis, lacunar cerebral infarction, chronic hepatitis B, cholecystitis, chronic bronchitis and other diseases, thus increasing the susceptibility of patients and the severity of the disease, so patients with these underlying diseases should be very closely monitored.

Some studies have shown that SARS-COV-2 is mainly transmitted by respiratory droplets and close contact [16], [17]. The epidemiological investigation of this outbreak showed that many individuals with COVID-19 were present in the community prior to this COVID-19 outbreak at our hospital, and several RT-PCR nucleic acid-positive specimens were detected among outpatients from the community. These RT-PCR-positive outpatients moved between the outpatient and inpatient units, and person-to-person transmission by respiratory droplets was ongoing. Consequently, infected patients on the ward could also transmit COVID-19 to the other inpatients through close contact. In addition, the Omicron variant has become the predominant strain in China and elsewhere. Information obtained from the Wenzhou Center for Disease Control and Prevention shows that the locally prevalent SARS-COV-2 strain is the omicron strain, which is highly transmissible [18], [19]. This strain caused an 84.5% attack rate among hospitalized patients in the present COVID-19 outbreak. After the first few cases occurred in some wards, COVID-19 quickly spread to other patients in the same wards, resulting in cross-contamination. All wards were eventually affected, and in some, all became ill. In addition, the Omicron strain spreads rapidly [20]; the number of cases in the UK increased exponentially every 2–3 days [18]. At the peak of the current outbreak, 482 people became ill in just 4 days, representing 63.3% of the total number of cases. Thus, this outbreak caused by the Omicron strain is not only highly transmissible but also rapidly spreading. The causative agent of this outbreak was identified as Omicron strain BF.5 by the Wenzhou Center for Disease Control and Prevention.

In this COVID-19 outbreak, 20 patients died, i.e., a case fatality rate of 2.62%, because they had not been previously vaccinated. The vast majority of infected patients were unvaccinated and some had multiple comorbidities. As a result, these patients were highly susceptible due to their lack of immunity or decreased resistance to viral infection; once infected, they were often prone to severe illness and death [21]. Studies have shown that the fatal cases were mainly elderly, high-risk individuals with underlying diseases [22]. Our survey showed that the deceased in this COVID-19 outbreak were older people. Of these deaths, 80% were over 70 years, and 45% were over 80 years of age. Most of these nucleic acid-positive deaths confirmed by RT-PCR were due to pulmonary infections, while a few patients died of sudden death, uremia, and gastrointestinal bleeding. Therefore, priority should be given to improving the protection of the elderly during COVID-19 outbreaks, especially the lonely elderly and vulnerable elderly with comorbidities. Therefore, adequate medical resources should be provided for these high-risk groups, and hospitals should be well prepared for the comorbidity and mortality of these elderly people as a result of COVID-19 infection. 

Most of the patients in the special care ward are elderly widows who have been hospitalized for a long time. Since their families rarely take care of them for various reasons, these patients mainly live on financial support from the government. The current survey showed that they did not receive COVID-19 vaccinations in the local community in the time before hospitalization, and thus their vaccination rate was low. Among the 284 cases hospitalized for special care, most were unvaccinated; only 2 cases were vaccinated against COVID-19, with a vaccination rate of 0.70%. These vulnerable populations should be vaccinated with a safe and effective COVID-19 vaccine in the future to improve their immunity.

## Limitations

Since this survey was an observational survey of a public health emergency, there will be some limitations. During this investigation, RT-PCR nucleic-acid testing or SARS-CoV-2 antigen examination was not performed on individuals without clinical symptoms. Some of these cases without clinical symptoms may actually be infected, which may also play an important role in the transmission process. In addition, the local vaccinations against COVID-19 prior to the outbreak were Sinovac-CoronaVac vaccine and SinoPharm-Beijing BBIBP-CorV vaccine. When the COVID-19 vaccination of patients was surveyed, it was difficult to distinguish which COVID-19 vaccine was administered to the recipients.

## Conclusion

Our investigation showed that the outbreak of COVID-19 among hospitalized patients in a psychiatric hospital had the characteristics of high incidence, strong infectivity, and rapid transmission at the end of 2022, when the government of China adjusted and optimized the COVID-19 prevention and control policy. Infectious sources were introduced into the hospital from the community and spread within the hospital, resulting in an outbreak. In the future, special attention should be paid to the elderly with underlying diseases when treating patients with COVID-19.

## Notes

### Competing Interests 

The authors declare that they have no competing financial interests or personal relationships that could have appeared to influence the work reported in this paper.

### Acknowledgements 

We would like to express out heartfelt gratitude to all staff at The Affiliated Kangning Hospital of Wenzhou Medical University who were involved in the investigation.

### Funding

This work was supported by 2022 Ministry of Education of China Humanities and Social Science Youth Foundation Project (22YJC790189); Shanghai Key Laboratory of Urban Design and Urban Science, NYU Shanghai Open Topic Grants (Grant No.2023YWZHOU_LOUD).

### Availability of data and materials 

The datasets used and analyzed in this study are available from the corresponding author on reasonable request. 

### Authors’ ORCID-ID

- Yiwei Zhou: 0000-0002-4625-6875
- Zumu Zhou: 0000-0002-5526-7808

## Figures and Tables

**Table 1:**
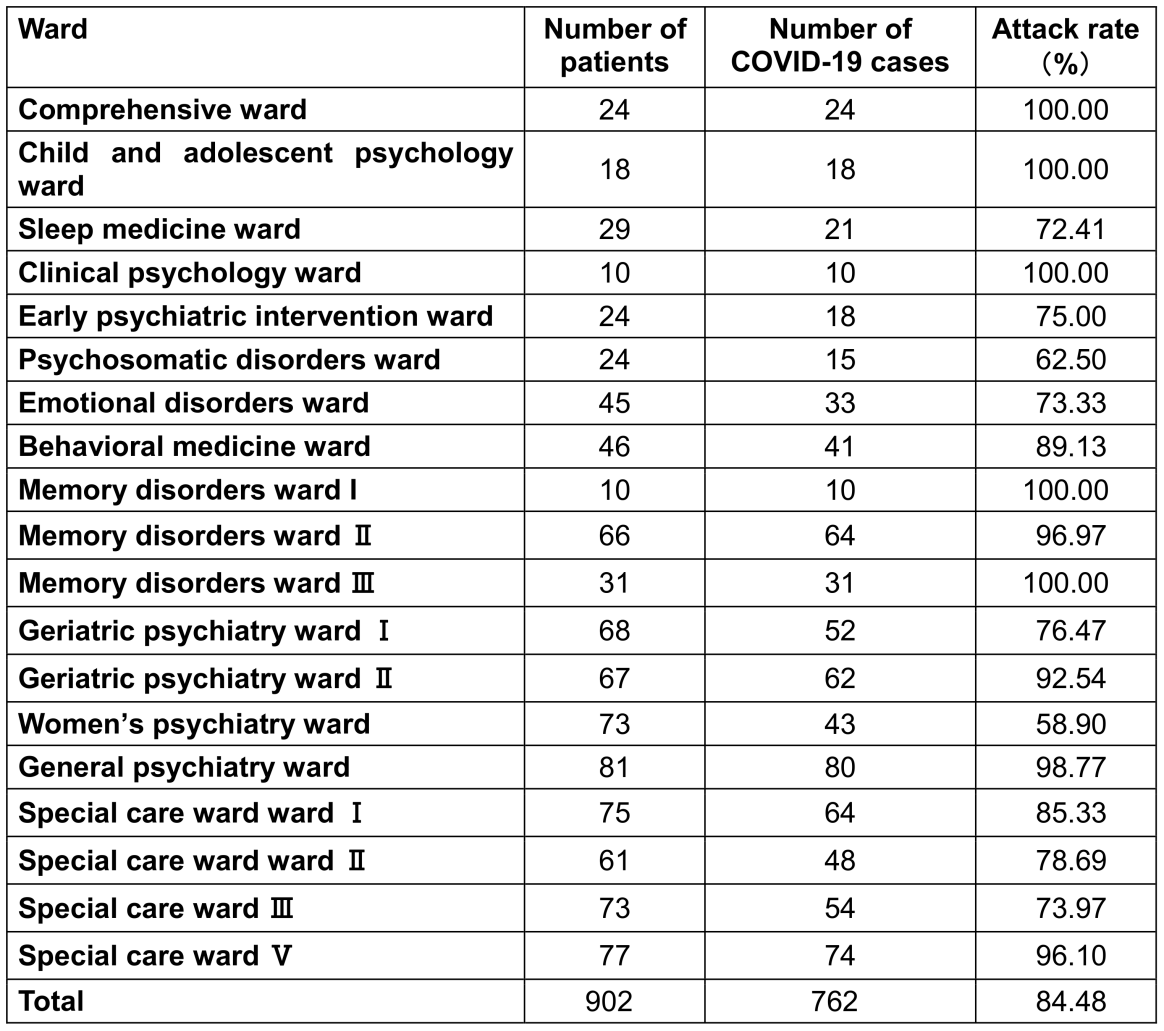
COVID-19 attack rate among inpatients by ward

**Table 2:**
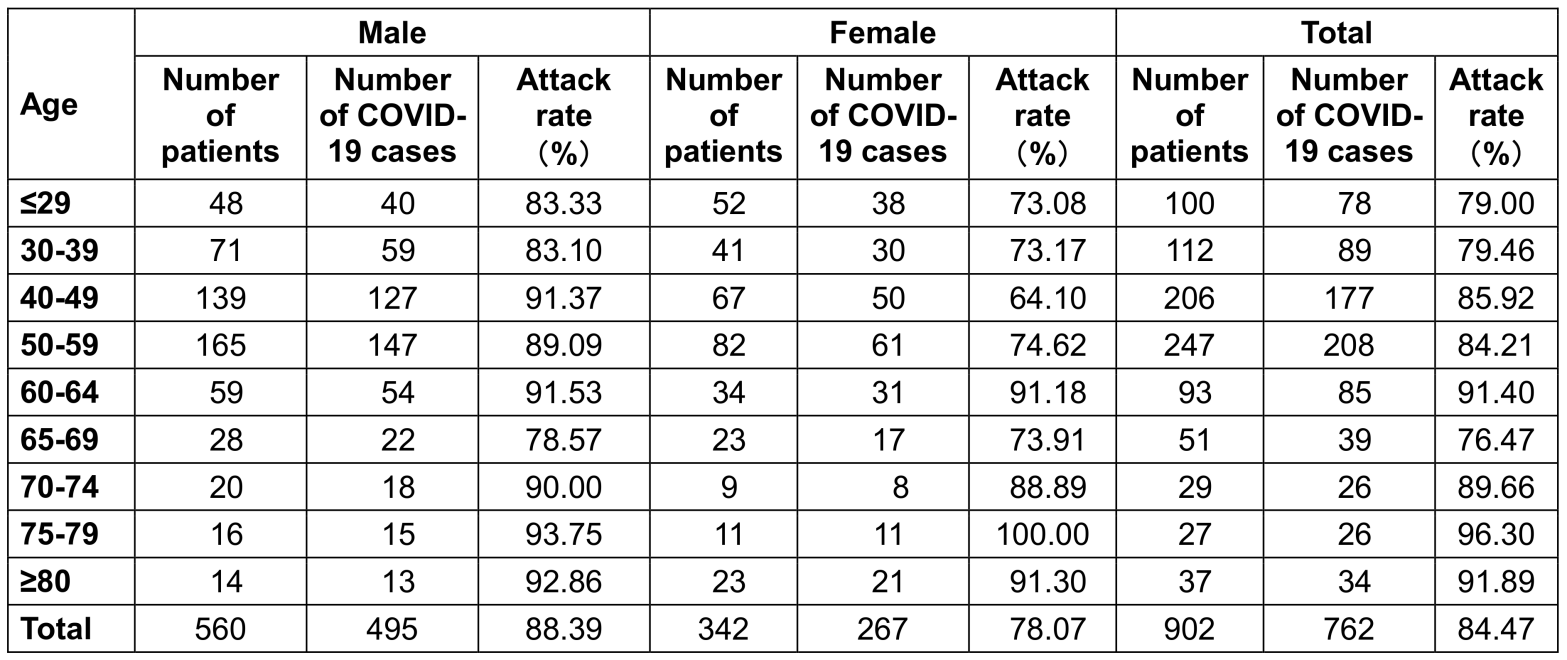
Attack rate of COVID-19 by gender and age

**Table 3:**
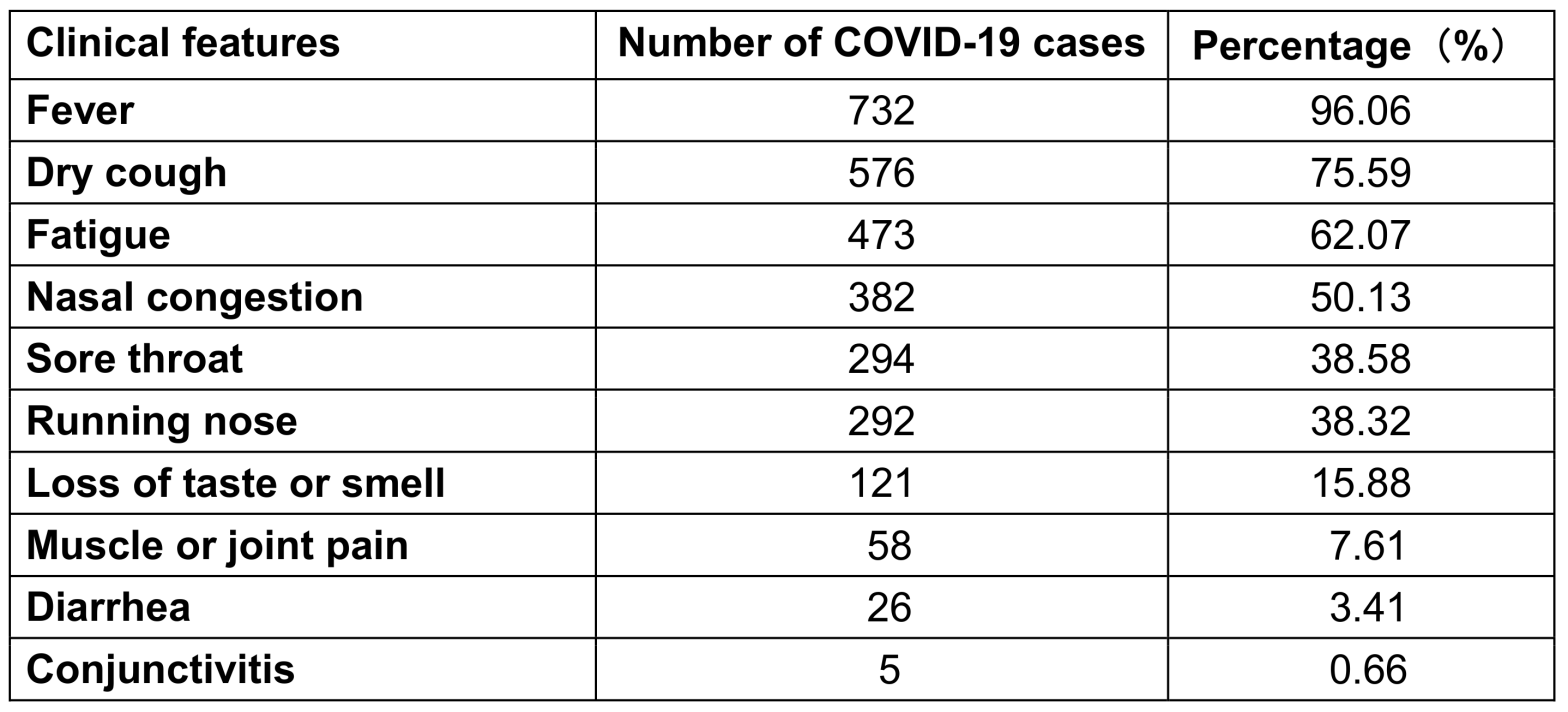
Proportion of various clinical manifestations of 762 COVID-19 cases

**Table 4:**
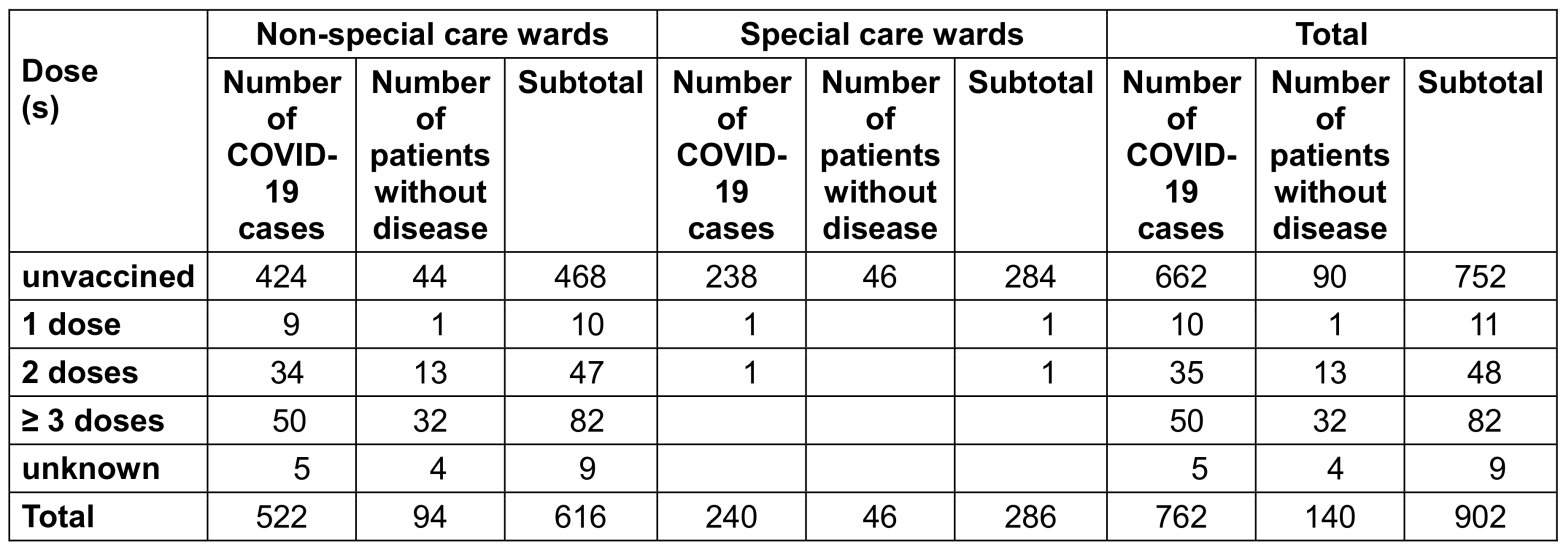
Vaccination rate of COVID-19 patients by ward
